# Characterization and Comparison of *Enterococcus* spp. Isolates from Feces of Healthy Dogs and Urine of Dogs with UTIs

**DOI:** 10.3390/ani11102845

**Published:** 2021-09-29

**Authors:** Dagmara Stępień-Pyśniak, Fabrizio Bertelloni, Marta Dec, Giulia Cagnoli, Dorota Pietras-Ożga, Renata Urban-Chmiel, Valentina Virginia Ebani

**Affiliations:** 1 Department of Veterinary Prevention and Avian Diseases, Faculty of Veterinary Medicine, University of Life Sciences in Lublin, Głęboka 30, 20-612 Lublin, Poland; dagmara.stepien@up.lublin.pl (D.S.-P.); marta.dec@up.lublin.pl (M.D.); renata.urban@up.lublin.pl (R.U.-C.); 2 Department of Veterinary Science, University of Pisa, 56124 Pisa, Italy; g.cagnoli@studenti.unipi.it (G.C.); valentina.virginia.ebani@unipi.it (V.V.E.); 3 Department of Epizootiology and Clinic of Infectious Diseases, Faculty of Veterinary Medicine, University of Life Sciences in Lublin, Głęboka 30, 20-612 Lublin, Poland; dorota.ozga@up.lublin.pl

**Keywords:** dog, *Enterococcus faecalis*, *Enterococcus faecium*, UTI, stools, antimicrobial resistance, biofilm, virulence

## Abstract

**Simple Summary:**

Infections caused by *Enterococcus* spp. represent a serious threat to human and animal health due to difficulties in treatment. Indeed, these bacteria are a very able “trafficker” of antimicrobial resistance genes and for this reason they are often resistant to many antimicrobials. In this study we explored the role of pet dogs as possible carriers and targets of antimicrobial resistant and virulent enterococci. Isolates collected from feces of healthy animals and urine of dogs suffering with UTIs were characterized and compared. Strains resulted as resistant to many of the antimicrobials tested and almost of them were multidrug-resistant. Diffuse resistance was recorded for compounds routinely employed in human and pet therapy. Genes responsible for antimicrobial resistance were widely detected. *E. faecalis* and *E. faecium* resulted as equally distributed in stool samples, while *E. faecalis* prevailed among UTI isolates; virulence genes were more often detected in bacteria belonging to this species. Our data confirm that enterococci inhabitant of the gut flora probably represent the main source of UTI in dogs. Furthermore, healthy and sick pet dogs could be spreaders of antimicrobial and virulent enterococci, representing a possible hazard for other animals and owners.

**Abstract:**

*Enterococcus* spp. are opportunistic pathogens of both humans and animals characterized by high resistance to antimicrobials. Dogs could be intestinal carriers or suffer from *Enterococcus* infections, mainly urinary tract infections (UTIs). This study aimed to analyze and compare *Enterococcus* spp. isolated from healthy dog stools and sick dog urine. Overall, 51 isolates (29 from stools and 22 from UTI) were characterized at species level and tested for antimicrobial resistance, biofilm production and presence of resistance and virulence genes. *E. faecium* and *E. faecalis* resulted as equally distributed in stools samples, while *E. faecalis* predominated among UTI isolates. HLAR phenotype was detected in 47.1% isolates; 64.7% isolates were resistant to ampicillin (47.1% with a MIC ≥ 64 µg/mL). High levels of resistance were recorded for fluoroquinolones (enrofloxacin 74.5%, ciprofloxacin 66.7%), clindamycin (84.3%), tetracycline (78.4%) and quinupristin–dalfopristin (78.4%). No vancomycin resistant strains were detected. All but one isolate were multidrug-resistant. Most detected resistance genes were *tetM* (70.5%), *pbp4* (52.9%) and aph(3′)-IIIa (39.2%). All isolates were able to produce biofilm, but isolates from UTIs and belonging to *E. faecalis* more frequently resulted in strong biofilm producers. Most detected virulence genes were *asa1* (52.9%), *gelE* (41.2%), *cylA* (37.3%) and *esp* (35.3%); all of them resulted as more frequently associated to *E. faecalis*. No particular differences emerged between isolates from feces and UTI, considering all evaluated aspects. Our results confirm pet dogs as carriers of multidrug-resistant enterococci; stool microflora could be considered as the most probable source of enterococcal UTI and *E. faecalis* carried by dogs seems to be more virulent than *E. faecium*, justifying its more frequent involvement in urinary tract infections.

## 1. Introduction

Bacteria belonging to *Enterococcus* genus are Gram-positive, catalase-negative, facultative anaerobic and non-spore-forming. Enterococci are commensal bacteria living in the gastrointestinal tract of many animals, from invertebrates to humans. More than 50 species have been described, but *Enterococcus faecium* and *Enterococcus faecalis* are the most detected in both humans and animals [1]. These bacteria are very hardy organisms; indeed, they can survive for months in the environment, tolerate different adverse conditions and endure host defenses [1,2]. For these reasons, enterococci are considered opportunistic pathogens in humans as well in animals and they are often causes of nosocomial infections [3]. The main problem associated with enterococcal infections is antimicrobial resistance. On one hand enterococci are intrinsically resistant to some antimicrobials; on the other hand they are very able to acquire and transfer resistant genes from other bacteria via plasmids and/or transposons [2,4]. The selective pressure linked to widespread use of antimicrobials drives an accumulation process of resistance genes in these bacteria and the selection of multidrug-resistant strains. As consequence, antimicrobial therapy of *Enterococcus* infection in humans need to be constantly modulated and changed [5]. One of the main raised problems was the resistance to vancomycin, considered as last line therapy [1]. Another example is the acquisition of genes conferring high level resistance to streptomycin or gentamicin and the magnitude of minimum inhibitory concentrations for ampicillin that removed the possibility of taking advantage of the synergism between these two compounds for therapy [2,5,6].

Dogs can be affected by enterococci and can act as reservoirs of virulent and multidrug-resistant strains. Different authors stressed that healthy dogs could be asymptomatic carriers of *Enterococcus* spp. at the intestinal level and *E. faecalis* and *E. faecium* are the most abundant species detected [3,7,8,9]. Vancomycin Resistant Enterococci (VRE) are rarely detected in pet healthy dogs [4,9,10], however, resistant and multidrug-resistant strains are frequently isolated [8,11,12]. As well as in humans, enterococci could be responsible of nosocomial infections in dogs [13]; nevertheless, the most common pathological condition associated with *Enterococcus* is urinary tract infection (UTI). UTI occurs as consequence of local host defenses alteration and/or invasion by virulent microorganisms, generally bacteria [14]. Most UTIs are ascending infections caused by rectal, perineal and genital flora [15]. Although *Escherichia coli* is the main pathogen isolated from UTIs in dogs, about 25% of canine infections are caused by Gram-positive cocci [16] and recent investigations suggest that *Enterococcus* species are involved in a percentage of cases between 3 and 11% [17,18,19,20].

The aim of this study was to characterize and compare *Enterococcus* spp. isolated from healthy dog feces and from dogs suffering from UTI. In particular, species involved, phenotypic and genotypic antimicrobial resistance, as well as multidrug-resistance, biofilm production and virulence of the selected isolates were investigated.

## 2. Materials and Methods

### 2.1. Bacteria Strains Provenience and Identification

The study was carried out on 51 enterococci that were previously isolated and kept in biobank of Infectious Disease Laboratory of Department of Veterinary Sciences, University of Pisa. In particular, 22 isolates were cultured from urine of dogs suffering UTIs, while 29 were from healthy dog fecal samples subjected to the laboratory for routine controls. Urines and stools samples were collected during 2019 from dogs living in Tuscany, Central Italy. Enterococci were isolated on Kanamycin Aesculin Azide Agar (KAAA) (Oxoid Ltd., Basingstoke, United Kingdom) and confirmed to genus, and presumptively species level by API 20Strep^®^ (Biomerieux, Marcy l’Etoile, France), during the routine clinical activities; all isolates were kept at −80 °C in Brain Heart Infusion (BHI) broth (Oxoid Ltd.) using glycerol as cryoprotectant.

Bacteria species was evaluated using an Ultraflextreme Matrix Assisted Laser Desorption Ionization Time of Flight Mass Spectrometry (MALDI-TOF MS) and MALDI-Biotyper 3.0 software (Bruker Daltonics, Bremen, Germany) as described previously [21]. The MALDI Biotyper output is a log (score) between 0 and 3.0, which is calculated from a comparison of the peak list from an unknown isolate with the reference main spectra in the database. According to the criteria proposed by the manufacturer, a log (score) below 1.700 does not allow for reliable identification; a log (score) between 1.700 and 1.999 allows identification to the genus level; a log (score) between 2.000 and 2.299 means highly probable identification at the genus level and probable identification at the species level and a log (score) higher than 2.300 (2.300–3.000) indicates highly probable identification at the species level. 

Phenotypic identification was confirmed with genotypic method, using primers and protocols previously reported. Species-specific primers were selected for the identification of *E. faecalis* (*E. faecalis*-F: ATCAAGTACAGTTAGTCTTTATTAG; *E. faecalis*-R: ACGATTCAAAGCTAACTGAATCAGT), *E. faecium* (*E. faecium*-F: TTGAGGCAGACCAGATTGACG and *E. faecium*-R: TATGACAGCGACTCCGATTCC) and *E. gallinarum* (*E. gallinarum*-F: TTACTTGCTGATTTTGATTCG and *E. gallinarum*-R: TGAATTCTTCTTTGAAATCAG) [22,23]. From overnight culture, the genomic DNA was extracted using the commercial Genomic Mini kit (A&A Biotechnology, Poland); extracted DNA was kept at 4 °C and employed for all the molecular investigations described subsequently. Uniplex PCRs were conducted using a DNA Mastercycler (Eppendorf, Germany) at following conditions: an initial denaturation at 95 °C for 4 min, followed by 30 cycles of denaturation at 95 °C for 30 s, annealing at 54 °C (*E. faecalis* and *E. faecium*) or 55 °C (*E. gallinarum*) for 1 min, extension at 72 °C for 1 min; a final extension step at 72 °C for 8 min was added. The reaction mixture was composed of 1 µL (~20 ng) of DNA as a template, 12.5 µL of 2X Taq PCR Master Mix (Qiagen, Poland), 1 µL of each of the two primers (10 pmol/µL, Genomed, Poland) and RNAse-free water (Qiagen, Poland) to a final volume of 20 µL. PCR products were resolved by electrophoresis on a 1.5% agarose gel stained with GelView (Novazym, Poland), and then visualized using a gel imaging analysis system with Quantity One software (Bio-Rad Laboratories, USA). The product lengths for the expected species of enterococci were 941bp for *E. faecalis*, 658bp for *E. faecium* and 173bp for *E. gallinarum*. Positive/negative controls for PCR were *E. faecalis* ATCC 51299 (vanB+), *E. faecium* ATCC 51559 (vanA+) and *E. gallinarum* ATCC 49573 (vanC1+).

### 2.2. Phenotypic Resistance

Disk diffusion method was employed to test the resistance of selected isolates to 21 antimicrobials, following CLSI guidelines [24]. The tested antimicrobial disks (Oxoid Ltd., Hampshire, United Kingdom) were: amoxicillin–clavulanic acid (30 μg), ampicillin (10 μg), cefalotin (30 μg), chloramphenicol (30 μg), ciprofloxacin (5 μg), clindamycin (2 μg), enrofloxacin (5 μg), erythromycin (10 μg), gentamicin (10 μg), linezolid (30 μg), neomycin (10 μg), nitrofurantoin (300 μg), oxacillin (1 μg), quinupristin–dalfopristin (15 μg), rifampicin (30 μg), streptomycin (10 μg), teicoplanin (30 μg), tetracycline (30 μg), tigecycline (15 μg), trimethoprim (5 μg),and vancomycin (30 μg). Results were interpreted following CLSI and EUCAST breakpoint tables [25,26]. Isolates resistant to streptomycin or gentamicin in disk diffusion test were examined for high level aminoglycoside resistance (HLAR) following CLSI guidelines [26] and employing high concentration of gentamicin (500 μg/mL) and streptomycin (1000 μg/mL). Minimum Inhibitory Concentration (MIC) for vancomycin and ampicillin was evaluated in all disk diffusion test resistant and intermediate isolates [26,27]. 

On the basis of phenotypic resistance patterns, isolates were classified as multidrug-resistant (MDR), extensively drug-resistant (XDR) or pandrug-resistant (PDR) [28].

### 2.3. Genotypic Resistance

To detect resistance genes and Tn916/Tn1545-like transposon (integrase gene Int-Tn), 26 gene-specific PCR primer pairs were used (Table S1). PCR mixtures and conditions for detection of tested genes were carried out following previously described protocols provided in the (supplementary materials Table S1).

The PCR products obtained for *mef*(A/E) gene were sequenced to confirm that the amplicons are counterparts of the resistance gene by comparative analysis using the NCBI BLAST algorithm; sequences were deposited in the GenBank database.

PCR products for *pbp5* genes were sequenced to assess the type of mutation and possible relationships in relation to the MIC values for ampicillin. Amino acid sequences of *pbp5* gene were compared with the *pbp5* protein reference sequences of *E. faecium* (GenBank Accession No. CAA59287.1). The nucleotide sequence for each gene was analyzed using MEGA X software, and phylogenetic UPGMA (unweighted-pair group method using average linkages), tree was constructed for *pbp5* gene.

### 2.4. Biofilm Production 

Biofilm production was evaluated as reported by Hashem et al. [29] and Bertelloni et al. [30], with some modifications. Isolates were growth overnight at 37 °C in Tryptic Soy Broth (Oxoid Ltd.) supplemented with 1% glucose (TSB-1%G). Cultures were adjusted to 0.5 McFarland density, diluted 1:100 in TSB-1%G and transferred in a sterile flat-bottomed 96-well polystyrene microtiter plates; three wells were employed for each isolate. Microtiter plates were incubated at 37 °C for 24 h, washed with sterile distilled water, dried at ambient temperature and stained with 0.1% crystal violet; subsequently, the dye was carefully removed and each well washed and dried as reported above. Bounded crystal violet was resuspended with 30% glacial acetic acid, and the 570 nm optical density (OD570) was read. Biofilm production was evaluated in 3 repeated independent experiments for each isolate. TSB-1%G alone was used as negative control and the optical density measured (ODc), was used to classify the strains: OD ≤ ODc = non-biofilm producers, ODc < OD < 2ODc = weak biofilm producers; 2ODc < OD < 4ODc = moderate biofilm producers and OD > 4ODc = strong biofilm producers [29].

### 2.5. Virulence Genes Detection

The virulence genes *asa1*, *esp*, *efaAfs*, *efaAfm*, *gelE*, *hyl*, *cylA*, *sgrA*, *pstD*, *orf1481* and *IS16* were searched using primers and protocols previously reported in literature and included in the (supplementary materials Table S2).

### 2.6. Clustering Analysis

Safety traits (antibiotic and virulence genes) of tested isolates were examined by cluster analysis with the NTSys ver. 2.02 program (Exeter Software Ltd.). Safety traits were recorded as ‘1′ or ‘0′ if positive or negative, respectively. The similarity distance between gene patterns was calculated using the Dice coefficient and the dendrogram was based on the UPMGA.

### 2.7. Statistical Analysis

The obtained data were analyzed using Chi-square (X^2^) test. Statistical tests were used to evaluate the difference between isolates from stools and urine in relation to phenotypic and genotypic antimicrobial resistance, biofilm production and in relation to presence of virulence genes. The statistical significance threshold was set at *p*-value ≤ 0.05.

## 3. Results

### 3.1. Bacteria Strains, Provenience and Identification

Based on identification with the use of the MALDI-TOF MS technique, 30 isolates were classified as *E. faecalis* (14 from healthy dog stools and 16 from sick dog urine), 18 isolates as *E. faecium* (15 from feces and 3 from urines) and 3 isolates as *E. gallinarum* (all from urines). MALDI-TOF MS analysis correctly identified 23 isolates with log (score) values of ≥ 2.3, which indicated ‘highly probable species identification’. In the case of 28 isolates, MALDI-TOF MS gave secure genus identification and probable species identification with a log (score) value of 2.025–2.290. MALDI-TOF MS confirmed biochemical identification of most isolates, with the exception of three *E. faecium* that were reclassified as *E. faecalis*, three *E. faecium* that were reclassified as *E. gallinarum* and two *E. durans* that were reclassified as *E. faecium*. Molecular identification confirmed MALDI-TOF MS results. Identification results obtained with MALDI-TOF MS were considered in the rest of this manuscript.

### 3.2. Phenotypic Resistance

Considering the results of disk diffusion test, high levels of resistance were detected for aminoglycosides (streptomycin 94.1%, neomycin 90.2%, gentamicin 68.6%), fluoroquinolones (enrofloxacin 74.5%, ciprofloxacin 66.7%), oxacillin (98%), clindamycin (84.3%), tetracycline (78.4%) and quinupristin–dalfopristin (78.4%). Meanwhile, against glycopeptides (vancomycin 13.7%, teicoplanin 3.9%) and amoxicillin/clavulanic acid (11.8%) few of tested isolates resulted as resistant. Most effective compounds included teicoplanin, 84.3% susceptible isolates, amoxicillin–clavulanic acid, 76.5% susceptible isolates and nitrofurantoin, 64.7% susceptible isolates. Table 1 reported detailed data on phenotypic resistance evaluation of all tested strains, in relation to kind of sample, sick dog urines or healthy dog feces. Some differences emerged; in particular, higher resistance was recorded for chloramphenicol, linezolid, nitrofurantoin, trimethoprim and tigecycline in isolates from stools, while isolates from urine were more resistant to quinupristin–dalfopristin, clindamycin and amoxicillin/clavulanic acid (*p* < 0.05).

A Minimum Inhibitory Concentration test for vancomycin did not confirm resistance in assessed isolates (MIC ≥ 32 µg/mL) and, consequently, all tested enterococci were considered phenotypically susceptible.

Minimum Inhibitory Concentration test for ampicillin confirmed disk diffusion results in all resistant isolates; furthermore, all but three intermediate isolates resulted resistant, presenting a MIC value over the breakpoint (≥ 16 µg/mL). Overall, considering MIC results, 33/51 (64.7%) isolates were ampicillin resistant. However, 24/33 (72.7%) resistant isolates showed a MIC ≥ 64 µg/mL. No statistical differences emerged between isolates from stools and urines (*p* > 0.05). 

High Level Aminoglycoside Resistance was detected in 24/51 (47.1%) isolates. In detail, 11 isolates shoved only high-level streptomycin resistance (HLSR), 3 isolates only high-level gentamicin resistance (HLGR) and 10 isolates showed a high level of resistance to both streptomycin and gentamicin. No statistical differences were observed between isolates from stools and urine (*p* > 0.05).

Nine isolates showed HLAR in combination with ampicillin MIC ≥ 64 µg/mL: 1 *E. faecalis* and two *E. faecium* from stools (all HLSR), two *E. faecalis* (all HLSR), two *E. faecium* (one HLGR and one resistant to both streptomycin and gentamicin) and two *E. gallinarum* (one HLSR and one resistant to both streptomycin and gentamicin) from urine.

Regarding multidrug-resistance, 44 isolates resulted MDR and 6 XDR; 1 *E. faecium* isolate did not result as multidrug-resistant. XDR isolates were detected only from healthy dog stools; this condition resulted as significantly associated with isolates from this kind of sample (*p* > 0.05).

Table 2 summarizes data on HLAR, ampicillin MIC and multidrug-resistance classification.

### 3.3. Genotypic Resistance

The genes *vanA*, *vanB*, *blaZ*, *tetO*, *tetK*, *ermA*, *aph(2”)-Ib*, *aph(2”)-Ic*, *aph(2”)-Id* and *ant(4’)-Ia* were not detected. The other searched resistance genes were found in at least one isolate (Table 3). Most detected ones were *tet*(M) (36/51; 70.5%), *pbp4* (27/51; 52.9%) and *aph(3′)-IIIa* (20/51; 39.2%). Furthermore, *Int-Tn*, part of transposons Tn*916*/Tn*1545*, was identified in 28/51 (54.9%).

Some genes responsible for resistance to aminoglycosides (*aph(3′)-IIIa*, *aac(6′)-Ii*, *ant(6’)-Ia*), macrolides (*mrsA/B*, *mrsC*) and lincosamides (*lnuB*) were more often associated with isolates from stools; while, the gene *pbp4* related to β lactams resistance was detected more frequently in isolates from sick dog urine (*p* > 0.05). No statistical differences emerged about the distribution of other genes. 

*mef*(A/E) gene was detected in *E. faecium* isolate from healthy dog feces and the nucleotide sequence of the product obtained for this gene has been deposited in GenBank under accession number: MN548247.

**Figure 1:**
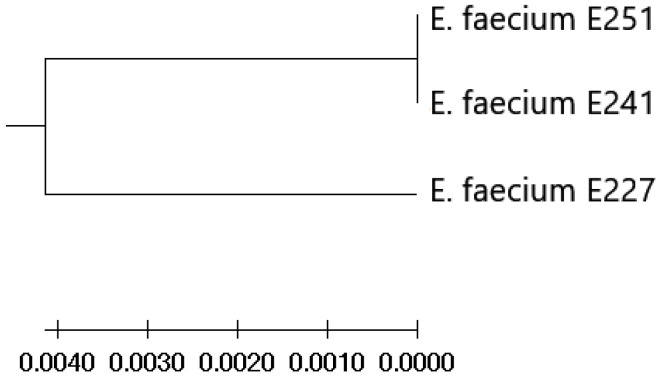
*Pbp5* phylogeny coincides with MIC value. An UPGMA tree of *pbp5* was constructed using the amino acid sequences from three studied isolates. *Pbp5* divides isolates into two groups based on amino acid sequence, and these two groups coincide with the MIC value. *E. faecium* isolate with an ampicillin MIC of 128 µg/mL was clustered separately from two *E. faecium* isolates with an MIC of 256 µg/mL in the tree.

Sequencing of the C-terminal region of *pbp5* gene revealed mutation responsible for amino acid changes at positions 633 E→R, 634 N→T in all studied isolates positive for *pbp5*. In addition, amino acid changes were found in the positions 471 V→I and 487 Q→L in E227 *E. faecium* isolate (MIC value of 256 µg/mL), but any changes in the same positions were observed for E251 and E241 *E. faecium* isolates (MIC values of 128 µg/mL) with respect to reference sequence of *E. faecium* (GenBank Accession No. CAA59287.1) (Figure 1).

### 3.4. Resistance Profiles: Comparison of Phenotypic and Genotypic Results

Regarding HLAR, among the 21 isolates showing high level streptomycin resistance, only 14 harbored the related resistance gene (*ant(6’)-Ia*); furthermore, three isolates were *ant(6’)-Ia* positive, but did not result HLSR. On the other hand, only two isolates had the gene *aac(6’)-Ie-aph(2”)-Ia* conferring resistance to high level of gentamycin and only one resulted as phenotypically resistant. Twelve isolates resulted HLGR, but they scored negative for all searched genes.

Sixteen phenotypically ampicillin resistant isolates scored positive for gene *pbp4*, *pbp5* or both. While 17 phenotypically resistant isolates presented anyone of the genes associated with β-lactams resistance. Fourteen isolates were positive for the gene *pbp4* but resulted as susceptible to ampicillin and amoxicillin/clavulanic acid.

All but one *cat* positive isolates were resistant to chloramphenicol; however, 11 out of 20 phenotypically resistant isolates scored negative for resistance gene.

Considering resistance to tetracycline, 36 phenotypically resistant isolates were positive for resistant genes. In particular, 26 harbored *tet*(M) only, 9 *tet*(M) and *tet*(L) in association and 1 isolate *tet*(L) only. Furthermore, four isolates resulted resistant in a disk diffusion test, but scored negative for all searched genes and one isolate positive for *tet*(M) was susceptible to tetracycline. All 28 isolates positive for *Int-Tn* were positive for *tet*(M) and 4 isolates were also positive for *tet*(L).

Regarding erythromycin, 19 resistant isolates were positive for one or more resistance genes (*erm*(B), *mrs*(A/B), *mrs*(C), *mef*(A/E)), whereas 10 resulted negative for all investigated genes. Twelve isolates harboring one or more resistance genes were susceptible to erythromycin; among them, 10 were positive for the *mrs*(A/B) and *mrs*(C) genes, 1 for gene *mrs*(C) and 2 for *erm*(B) gene.

All isolates harboring *lnuB* gene resulted resistant to clindamycin. However, 32 resistant isolates scored negative to resistance gene.

Finally, all 40 quinupristin–dalfopristin resistant isolates were negative for both *vatE* and *vatD*.

Table S3 reports the phenotypic and genotypic profile of each analyzed isolates as well as data on kind of sample and bacterial species.

### 3.5. Biofilm Production

All the analyzed enterococci resulted as biofilm producers. In detail, 21/51 (41.2%) were classified as weak biofilm producers, 11/51 (21.5%) as moderate biofilm producers and 19/51 (37.3%) as strong biofilm producers. Table 4 reports the distribution of biofilm production in relation to origin of samples and bacterial species. A statistical difference (*p* < 0.05) was observed between isolates from stools and UTI: higher level of biofilm production was observed in enterococci from urines. Statistical differences (*p* < 0.05) emerged also in relation to species: *E. faecalis* resulted as a higher biofilm producer than other species.

### 3.6. Virulence Genes and Virulence Profiles

Most detected genes were the ones coding for species-specific surface adhesins, *efaA_fs_* and *efaA_fm_*, that were present in all investigated *E. faecalis* and *E. faecium* isolates, respectively. Other genes encoding for enterococcal surface protein, *esp*, and aggregation substance, *asa1*, were largely present in the investigated bacteria: 18/51 (35.3%) and 27/51 (52.9%) positive isolates, respectively.

Other frequently detected genes were *gelE*, 21/51 (41.2%), and *cylA*, 19/51 (37.3%), related to gelatinase and cytolysin production, respectively. With the exception of 3 *E. faecium* isolates positive for *gelE* and/or *cylA* and 1 *E. gallinarum* positive for *gelE*, these genes were detected mainly in *E. faecalis*.

The genes *sgrA*, *pstD* and *orf1481* were detected in less than 10% of isolates; meanwhile, *hyl* gene was not found in investigated enterococci.

Finally, the IS16 insertion element, often detected in human hospital strains, was detected in 10/51 (19.6%) isolates.

Table 5 reports the detailed distribution of virulence genes and data about phenotypic expression of *gelE* and *cylA*.

No statistical differences emerged about the distribution of virulence genes between isolates from healthy dog stools and urine of dogs with UTIs.

Considering the association of virulence genes, 18 different profiles were detected. All isolates were positive for at least one virulence gene. The number of genes found together in an isolate ranged between 1 and 7 (Table S3).

Finally, the genes *asa1*, *cylA* and *esp* more frequently resulted in strong biofilm producer strains (*p* < 0.05).

### 3.7. Clustering Analysis of Relevant Safety Traits

**Figure 2:**
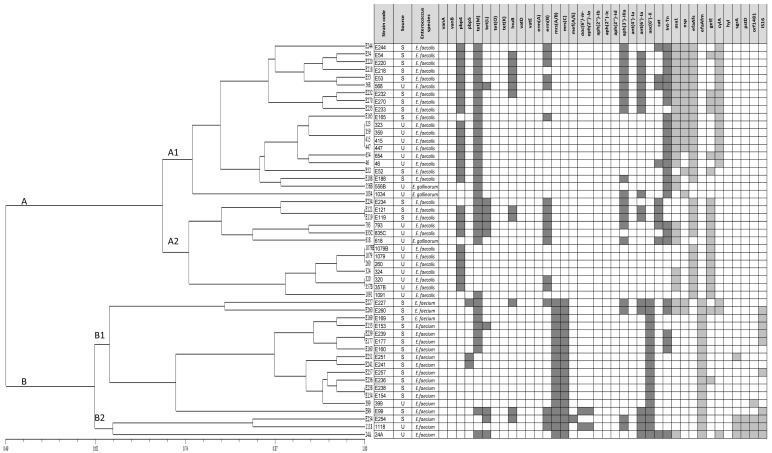
Dendrogram generated from safety traits data based on antibiotic and virulence patterns found in analyzed enterococcal isolates. The scale indicates the similarity level. The main groups clustering individual enterococcal species are designated A and B, while their subgroups linking isolates in response to certain genes are described A1, A2, B1 and B2, respectively. S—Stool, U—Urine.

The isolates were clustered in two main groups (A and B). Group A contained *E. faecalis* and *E. gallinarum* isolates. Subsequently, two subgroups (A1 and A2) were identified within group A, which accumulated isolates mainly based on the presence of the *tet*(M), *Int-Tn* (*Tn916/Tn1585*), *asa1*, *esp* and *cylA* (A1) and *gelE* (A2) genes, respectively. Group B contained only *E. faecium* isolates. This group also had two main subgroups (B1 and B2), which had combined isolates mainly based on the simultaneous presence (B2) or absence (B1) of the *sgrA*, *pstD*, *orf1481* and *IS16* genes. Furthermore, bifunctional (*aac(6′)-Ie-aph(2″)-Ia*) gene was found within one isolate belonging to subgroup B2 and in the E99 isolate adjacent to this subgroup in contrast to the *pbp5* gene, which was present in three isolates pooled within subgroup B1. Figure 2 shows details of the resistance and virulence gene map from which the similarity of the tested isolates was derived.

## 4. Discussion

In the last few years the opportunistic pathogens belonging to *Enterococcus* genus obtained a great deal of attention due to their frequent involvement in nosocomial infections linked to high level of antimicrobial resistance. Pets may act as reservoirs of multidrug-resistant enterococci, but may also be infected and develop disease. UTIs are the most frequent infection associated with *Enterococcus* infections in dogs [13].

In this study, isolates from two distinct kind of samples were analyzed under different points of view and compared. All isolates were collected in the same period of time from animals subjected to routine microbiological analysis. Urine samples were from dogs suffering UTI and *Enterococcus* spp. was diagnosed as the main responsible pathogen (generally the only one detected in pure culture). Stools from healthy dogs were subjected for routine feces control (presence/absence of *Salmonella* spp.). *Enterococcus faecalis* was the species most frequently detected, especially in UTI, with no statistical differences between the two kinds of sample. *E. faecium* resulted as more frequently present in feces (*p* < 0.05), and rarely involved in UTI. This is in line with other surveys, suggesting that *E. faecalis* is the primary species involved in enterococcal bacteriuria [31,32,33]. The two species were equally distributed in stools samples, as reported by other authors [3,7,9]. *E. gallinarum* is rarely reported as agent of canine UTIs [34], and is probably not a primary pathogen of dogs, as emerged from our investigation.

Antimicrobial resistance is probably the main threat in *Enterococcus* infections; indeed, these bacteria are frequently resistant to many antimicrobial agents [4]. In the present study, a very high percentage of isolates resulted resistant to aminoglycosides, fluoroquinolones, oxacillin, clindamycin, tetracycline and quinupristin–dalfopristin.

Aminoglycoside resistance in enterococci is well documented; indeed, in humans the therapeutic options are limited to streptomycin and gentamicin, only in combination with a cell-wall–active agent, commonly a β-lactam [6]. Generally, aminoglycoside resistance in enterococci from dogs is low [9,12,33,35,36], but some authors reported data similar to those obtained in the present study [11,37]. About half of resistant strains resulted HLAR and this is an atypical result; indeed, this condition is rarely reported in isolates from animals [12,38], with rare exceptions [39]. Furthermore, streptomycin and gentamicin are rarely employed in pet systemic therapy, due to their toxic effects, to justify a possible selection pressure.

No statistical differences were observed between stools and urine isolates, suggesting that this trait is probably fixed in *Enterococcus* population from this “ecosystem”. To strengthen this finding, the genes *ant(6’)-Ia* and *aac(6’)-Ie-aph(2”)-Ia* were largely detected in tested enterococci. Nevertheless, not all phenotypical resistant isolates scored positive to the investigated resistance genes; this result suggests other mechanisms of resistance, probably linked to other mobile elements or to genome mutations [4,5,6]. Furthermore, all analyzed *E. faecium* isolates had the *aac(6’)-Ii* gene, coding for a chromosomal aminoglycoside acetyltransferase specific for *E. faecium,* associated with the intrinsic low-level resistance to aminoglycosides, which was in agreement with other data [40,41].

In human therapy, ampicillin was generally used in combination with streptomycin or gentamicin to treat *Enterococcus* infections; resistance to high concentration of ampicillin (MIC ≥ 64 µg/mL) limits this possibility [2,5,6]. In our survey 51.0 and 19.6% of isolates resulted resistant and intermediate, respectively, to ampicillin in a disk diffusion method; all but three of them (64.7%) were confirmed as resistant in the broth microdilution assay. This percentage is higher than those observed in many other surveys on antimicrobial resistance in healthy and sick dogs [10,32,33,37,38,42]; however, some authors obtained similar results to those that emerged from our investigation [3,9,11]. A MIC value equal to or greater than 64 µg/mL was found in 72.7% of resistant isolates (47.1% of the total); this result stresses the hazard that these strains could represent for human health. The gene *pbp4* was the second most detected among resistance genes. However, its presence only partially explains the phenotypic results. The gene *pbp4* was found in both resistant and susceptible isolates and, among resistant strains, its presence was not associated to higher MIC values (*p* > 0.05). β-lactams resistance in *Enterococcus* is generally linked to the production of β-lactamase, to the overexpression of penicillin-binding proteins (PBPs) or to the alterations of PBPs, resulting in the production of PBPs with lower affinity with β-lactams. PBP4 and PBP5, expressed, respectively, by genes *pbp4* and *pbp5*, are generally involved in these two last types of resistance [43,44]. However, some studies showed that these genes could be present in both resistant and susceptible strains; indeed, ampicillin resistance is generally associated to point mutations in some specific position of these genes [45], which is consistent with our results for mutations in the *pbp5* gene.

Although the expression of *pbp4* gene, alone or in combination with other genes, could mediate cephalosporin resistance too [46], in our study, no relations were found between the resistance to cephalothin and the presence of *pbp4* and/or *pbp5*.

Even though the detected abundant ampicillin resistance, most of strains resulted as susceptible to amoxicillin/clavulanic acid, showing the potential effectiveness of this association; our data are in agreement to the results found in other studies [35,37,42], even if many authors reported a higher percentage of resistant enterococci in dogs, between 20 and 30% [11,32,33,39].

Tetracycline resistance was detected in 78.4% of isolates. This is a common finding, in agreement with other works on dogs reporting similar percentages in *Enterococcus* from feces [9,12,37,38,39] and other clinical specimens [11,33,42]. High level of tetracycline resistance are probably linked to the abundant use, in past years, of these antimicrobials, not only in pets, but more in general in animals and also in humans [47]. Molecular analyses support phenotypic results: tetracycline resistance genes were the most frequently detected genes and 90% of resistant isolates had *tet*(M), *tet*(L) or both genes, similarly to other studies [4,47]. The frequent detection (54.9%) of transposons Tn*916*/Tn*1545*, always in association with *tet* genes, underlines the possibility for these genes to jump from a strain to another and, consequently, the possibility for wide diffusion.

Resistance to oxacillin is well documented in enterococci and it could be considered as intrinsic [32,48,49,50]. Our data seem to confirm this statement, despite not all strains resulting resistant.

Resistance to clindamycin is considered intrinsic in *Enterococcus*, too; furthermore, this antimicrobial is considered ineffective in vivo also in case of in vitro susceptible isolates [4]. Our results are in line with this statement; however, it is interesting to note that clinical isolates more often resulted resistant. This could be related to the frequent use of this compound in pets therapy. The gene *lnuB*, conferring resistance to lincosamides and frequently carried by plasmids [51,52], was detected in relatively few isolates (21.6%), mainly *E. faecalis*, and it alone did not justify the detected level of resistance.

Resistance to quinupristin–dalfopristin is considered intrinsic only in some enterococcal species, in particular *E. faecalis* and *E. gallinarum* [26]. All analyzed *E. faecalis* and *E. gallinarum* isolates resulted resistant. A percentage of 38.8% of *E. faecium* were resistant, suggesting a high level of resistance in this species too, among tested isolates. These data are in line with other investigations [3,37]. None of the genes searched for antimicrobial resistance to streptogramins were detected; this finding suggests that other resistance mechanisms are involved in quinupristin–dalfopristin resistance among investigated isolates. In particular, efflux pumps encoded by macrolides resistance genes *msrA/B*, *mefA*, *lsa*, and *vga*, can confer resistance to streptogramins, too [53]. All *E. faecium* isolates analyzed in our study were positive for *mrs*(A/B) and *mrs*(C); one isolate was also positive for *mef*(A/E). Similarly, Aun et al. [40] noted that all *E. faecium* isolates had a chromosomal *msr*(C) gene associated with macrolide–streptogramin B resistance. In addition, Portillo et al. [41] found that PCR analysis with the *msr*(A)-specific primers designed for *Staphylococcus aureus* gives a DNA fragment of the expected size for all *E. faecium* strains, because it shows 62% identity at the nucleotide level with the *msr*(A) gene. This probably explains why the *E. faecium* isolates we tested as positive for the *msr*(C) gene were also positive for the *msr*(A/B) gene.

Erythromycin is not used for treatment of human enterococcal infections, but it is widely used in veterinary medicine, especially in production animals [54]. Percentage of resistance to this antimicrobial in *Enterococcus* strains from sick or healthy dogs is very variable, ranging between 10 and 80%, but it is generally high [3,9,32,33,37,38,39]. Results obtained in our study are in line with this research. Resistance to macrolide could be mediated by production of an enzyme that modifies ribosomal subunit, reducing the affinity of antimicrobial to ribosomes, or by efflux pumps; both of these events could be mediated by mobile genes such as *erm* or *mef* and *msr*, respectively [6,43]. The genes *erm(B)*, *msr(A/B)* and *msr(C)* were largely detected in tested strains; however there was not a strong association with phenotypic resistance and this is in agreement with some other studies [52,55,56,57].

A high level of resistance was detected for fluoroquinolones, both in sick and healthy dog samples. This is in line with results of other investigations [3,32,33,37,55], even if some authors reported lower percentages of resistant strains [9,38]. Quinolone resistance mainly occurs via point mutation of bacterial genes for DNA gyrase and topoisomerase IV [43]. Although enterococci have an intrinsic reduced susceptibility to fluoroquinolones, the extensive use of enrofloxacin in veterinary, mainly pet, therapy could have contributed to the spread of resistance [4].

A middle level of resistance was detected for the remaining antimicrobials, rifampicin chloramphenicol, linezolid, nitrofurantoin and trimethoprim, ranging between 25 and 60%. These compounds are not frequently used in veterinary medicine, but are sometimes employed in humans for treatment of different infections, including enterococcal ones [5,6,43,58]. Resistance to these antimicrobials in *Enterococcus* strains from pet dogs is very variable, as emerged from different studies [9,11,32,33,38,39].

Vancomycin resistant enterococci were not detected in this survey, with both phenotypic and genotypic methods; teicoplanin resulted effective with most tested isolates. These findings are in agreement with most other studies; indeed glycopeptides resistance in pet dogs, and more in general in animals, is generally low [4,9,38,39,55], with some exceptions [3,33].

The last aspect evaluated in relation to antimicrobial resistance was the concurrent resistance to more compounds. All but one isolate resulted as multidrug-resistant; in particular, 44 isolates were MDR and 6 were XDR, following Magiorakos et al.’s classification [28]. This output is in line with different studies on enterococci from dogs [11,33,37,39], confirming that these bacteria are frequently and significantly involved in this hazardous problem for pets and for public health. Antimicrobial resistance in *Enterococcus* is well documented in the literature and different authors explored the role of animals as reservoirs and sources of multidrug-resistant enterococci for humans [4,59]. However, a great variability has been observed in relation to geographic areas and context, animal species, type of samples and panel of tested antimicrobials. Considering that antimicrobial resistance is a phenomenon in evolution, its monitoring is necessary, mainly in pet dogs that often live in close contact with their owners.

Finally, virulence of isolates was evaluated by biofilm production assay and virulence gene detection. All isolates resulted as biofilm producers. However, isolates from UTI and belonging to *E. faecalis* showed a higher level of biofilm production. Biofilm production is considered a very important factor in the establishment of urinary tract infections. Indeed, biofilm helps bacteria to adhere to biotic, as well as abiotic, surfaces; furthermore, it protects them from being flushed by urine and from local body defense and antimicrobial substances [14]. Our data confirm the importance of biofilm production in the pathogenesis of UTI; furthermore, the higher level of biofilm production found in *E. faecalis* could explain why this species is more often isolated from urine samples. Our results are in agreement with data derived from human medicine: generally, *Enterococcus* isolates from UTI are biofilm producers and *E. faecalis*, the primary enterococcal species isolated from urinary tract infections, are stronger producer than other species [29,60]. In our investigation, the genes *asa1*, *cylA* and *esp* were found more frequently in strong biofilm producer strains, suggesting their involvement in biofilm production. However, contrasting data emerge from literature about the set of genes leading to biofilm formation in *Enterococcus* [29,60,61,62,63]; probably, the biofilm formation is a multifactorial process far from being fully understood.

All isolates presented the gene encoding surface adhesins specific for their own species. This is an expected result and data are in accordance with the available literature [12,64,65,66]. Excluding surface adhesins specific genes, the most detected genes were *asa1* (52.9%), *gelE* (41.2%), *cylA* (37.3%) and *esp* (35.3%). All these genes improve the ability of enterococci to enter and spread through the body. In particular, *asa1* encodes an aggregation substance that facilitates adhesion and sharing of plasmids, *gelE* codes for a gelatinase capable of hydrolyzing different peptides, *cylA* is part of an operon elaborating a hemolytic cytolysin and *esp* is associated with increased virulence linked to enhanced colonization and persistence in body districts and biofilm formation. The percentage of detection in our study was generally higher than in other surveys on pet dogs [12,35,67,68]. All these genes were more associated with *E. faecalis* than other investigated species (*p* < 0.05), and this is in line with the literature data [69]. The higher virulence of *E. faecalis* isolates could explain the higher detection in clinical samples. 

## 5. Conclusions

Data obtained in the present study confirm the double role of pet dogs as reservoirs and target hosts of enterococci. *E. faecalis* was confirmed as the primary species involved in canine UTIs. No relevant differences emerged between fecal and urinary isolates, with the exception of biofilm production. Although *E. faecalis* and *E. faecium* were equally present in stools, *E. faecium* resulted as less virulent and able to produce biofilm, and these features could explain its less frequent involvement in infections. Moreover, our results confirm the spread of antimicrobial resistance among enterococci, also in clinical isolates from pets. Antibiotic resistance is a severe concern for human and veterinary medicine. Canine enterococcal infections may be very difficult to treat; furthermore, infected dogs may be a source of *Enterococcus* spp. for their owners who, mainly in case of immunocompromised persons, can develop severe pathologies.

## Figures and Tables

**Table 1:** Results of disk diffusion antimicrobial test.

Antimicrobial		Resistant N (%)			Intermediate N (%)			Susceptible N (%)		
Class	Agent	Total	Stool	Urine	Total	Stool	Urine	Total	Stool	Urine
Ansamycins	RD	22 (43.1)	15 (51.7)	7 (31.8)	11 (21.6)	5 (17.2)	6 (27.3)	18 (35.3)	9 (31.0)	9 (40.9)
Phenicols	C	21 (41.2)	16 (55.2)	5 (22.7)	8 (15.7)	5 (17.2)	3 (13.6)	22 (43.1)	8 (27.6)	14 (63.6)
Oxazolidinones	LZD	21 (41.2)	21 (72.4)	0 (0.0)	5 (9.8)	3 (10.3)	2 (9.1)	25 (49.0)	5 (17.2)	20 (90.9)
Nitrofurantoins	F	13 (25.5)	13 (44.8)	0 (0.0)	5 (9.8)	3 (10.3)	2 (9.1)	33 (64.7)	13 (44.8)	20 (90.9)
Folate pathway antagonists	W	31 (60.8)	23 (79.3)	8 (36.4)	19 (37.3)	6 (20.7)	13 (59.1)	1 (2.0)	0 (0.0)	1 (4.5)
Aminoglycoside	N	46 (90.2)	28 (96.6)	18 (81.8)	4 (7.8)	1 (3.4)	3 (13.6)	1 (2.0)	0 (0.0)	1 (4.5)
	CN	35 (68.6)	20 (69.0)	15 (68.2)	4 (7.8)	2 (6.9)	2 (9.1)	12 (23.5)	7 (24.1)	5 (22.7)
	S	48 (94.1)	28 (96.6)	20 (90.9)	2 (3.9)	1 (3.4)	1 (4.5)	1 (2.0)	0 (0.0)	1 (4.5)
Cephems	KF	32 (62.7)	17 (58.6)	15 (68.2)	14 (27.5)	10 (34.5)	4 (18.2)	5 (9.8)	2 (6.9)	3 (13.6)
Fluoroquinolones	CIP	34 (66.7)	18 (62.1)	16 (72.7)	7 (13.7)	7 (24.1)	0 (0.0)	10 (19.6)	4 (13.8)	6 (27.3)
	ENR	38 (74.5)	22 (75.9)	16 (72.7)	9 (17.6)	5 (17.2)	4 (18.2)	4 (7.8)	2 (6.9)	2 (9.1)
Glycopeptides	TEC	2 (3.9)	2 (6.9)	0 (0.0)	6 (11.8)	3 (10.3)	3 (13.6)	43 (84.3)	24 (82.8)	19 (86.4)
	VA	7 (13.7)	2 (6.9)	5 (22.7)	15 (29.4)	7 (24.1)	8 (36.4)	29 (56.9)	20 (69.0)	9 (40.9)
Macrolides, Streptogramins, Lincosamides	E	29 (56.9)	16 (55.2)	13 (59.1)	17 (33.3)	11 (37.9)	6 (27.3)	5 (9.8)	2 (6.9)	3 (13.6)
	QD	40 (78.4)	19 (65.5)	21 (95.5)	7 (13.7)	6 (20.7)	1 (4.5)	4 (7.8)	4 (13.8)	0 (0.0)
	DA	43 (84.3)	21 (72.4)	22 (100.0)	6 (11.8)	6 (20.7)	0 (0.0)	2 (3.9)	2 (6.9)	0 (0.0)
Penicillins	OX	50 (98.0)	28 (96.6)	22 (100.0)	1 (2.0)	1 (3.4)	0 (0.0)	0 (0.0)	0 (0.0)	0 (0.0)
	AMC	6 (11.8)	1 (3.4)	5 (22.7)	6 (11.8)	6 (20.7)	0 (0.0)	39 (76.5)	22 (75.9)	17 (77.3)
	AMP	26 (51.0)	17 (58.6)	9 (40.9)	10 (19.6)	0 (0.0)	10 (45.5)	15 (29.4)	12 (41.4)	3 (13.6)
Tetracyclines	TE	40 (78.4)	24 (82.8)	16 (72.7)	5 (9.8)	2 (6.9)	3 (13.6)	6 (11.8)	3 (10.3)	3 (13.6)
	TIG	15 (29.4)	15 (51.7)	0 (0.0)	8 (15.7)	6 (20.7)	2 (9.1)	28 (54.9)	8 (27.6)	20 (90.9)

RD = rifampicin; C = chloramphenicol; LZD = linezolid; F = nitrofurantoin; W = trimethoprim; N = neomycin; CN = gentamicin; S = streptomycin; KF = cephalothin; CIP = ciprofloxacin; ENR = enrofloxacin; TEC = teicoplanin; VA = vancomycin; E = erythromycin; QD = quinupristin–dalfopristin; DA = clindamycin; OX = oxacillin; AMC = amoxicillin–clavulanic acid; AMP = ampicillin; TE = tetracycline; TIG = tigecycline.

**Table 2:** Multidrug-resistance, ampicillin MIC and high level aminoglycosides resistance detected, in relation to species and origin of samples.

Origin of Samples	Bacteria Species	Multidrug-Resistant Category		Ampicillin MIC ≥ 64 µg/mL	HLAR			HLAR in Isolates with Ampicillin MIC ≥ 64 µg/mL		
		MDR	XDR		HLSR	HLGR	Both	HLSR	HLGR	Both
Feces of healthy dogs	*E. faecalis*	11	3	1	12	10	8	1	0	0
	*E. faecium*	11	3	13	2	0	0	2	0	0
Urine of dogs with UTIs	*E. faecalis*	16	0	6	4	0	0	2	0	0
	*E. faecium*	3	0	2	1	2	1	1	2	1
	*E. gallinarum*	3	0	2	2	1	1	2	1	1
Total		44	6	24	21	13	10	8	3	2

**Table 3:** Distribution of resistance genes among *Enterococcus* spp. isolated from healthy and sick dogs.

Antibiotic	Resistance Gene	Healthy Dog Feces		Sick Dog Urine			Total *n* = 51
		*E. faecalis* *n* = 14	*E. faecium* *n* = 15	*E. faecalis* *n* = 16	*E. faecium* *n* = 3	*E. gallinarum* *n* = 3	
Gentamicin (HL)	*aac(6’)-Ie-aph(2”)-Ia*	0	1	0	1	0	2
Kanamycin (HL)	*aph(3′)-IIIa*	12	3	2	1	2	20
Gentamicin and other aminoglycosides (ML)	*aac(6′)-Ii*	0	15	0	3	0	18
Streptomycin (HL)	*ant(6’)-Ia*	9	4	1	2	1	17
Chloramphenicol	*cat*	5	0	3	1	1	10
β lactams	*pbp4*	12	0	15	0	0	27
	*pbp5*	0	3	0	0	0	3
Tetracyclines	*tet*(M)	14	8	10	1	3	36
	*tet*(L)	3	3	3	1	0	10
Lincosamides	*lnuB*	7	3	1	0	0	11
Macrolides	*erm*(B)	8	3	5	1	1	18
	*mrs(*A/B*)*	0	15	0	3	0	18
	*mrs*(C)	1	13	0	2	0	16
	*mef*(A/B)	0	1	0	0	0	1
-	*Int-Tn*	10	5	9	1	3	28

HL—High level; ML—Moderate level.

**Table 4:** Biofilm production by examined *Enterococcus* spp. isolates.

Sample	Species	WBP	MBP	SBP
Feces of healthy dogs	*E. faecalis*	1	3	10
	*E. faecium*	15	0	0
Urine of dogs with UTIs	*E. faecalis*	3	5	8
	*E. faecium*	2	1	0
	*E. gallinarum*	0	2	1
Total		21	11	19

Legend: WBP = Weak biofilm producers; MBP = Moderate biofilm producers; SB = Strong biofilm producers.

**Table 5:** Prevalence of virulence genes detected in *Enterococcus* spp. isolates.

Virulence Genes	Feces of Healthy Dogs		Urine of Dogs with UTIs			Total *n*= 51 (%)
	*E. faecalis* *n* = 14	*E. faecium* *n* = 15	*E. faecalis* *n* = 16	*E. faecium* *n* = 3	*E. gallinarum* *n* = 3	
*cylA*	9 (8 ^a^)	2 (2 ^a^)	7 (2 ^a^)	1 (1 ^a^)	0	19 (37.3)
*gelE*	8 (8 ^b^)	2 (1 ^a^)	10 (5 ^b^)	0	1	21 (41.2)
*asa1*	10	2	9	4	2	27 (52.9)
*esp*	10	2	5	0	1	18 (35.3)
*efaAfs*	14	0	16	0	0	30 (58.8)
*efaAfm*	0	15	0	3	0	18 (35.3)
*sgrA*	0	2	0	2	0	4 (7.8)
*pstD*	0	1	0	2	0	3 (5.9)
*orf1481*	0	1	0	3	0	4 (7.8)
*IS16*	0	8	0	2	0	10 (19.6)

^a^ = number of isolates positive for β-hemotytic activity; ^b^ = number of isolates showing gelatinase activity.

## Data Availability

The data presented in this study are contained within the article or supplementary material.

## Supplementary Materials

The following are available online at https://www.mdpi.com/article/10.3390/ani11102845/s1, Table S1: Primers of antibiotic resistance used for PCR in the study; Table S2: Target genes and primers used in this study; Table S3: Origin, species, phenotypic and genotypic profile of each *Enterococcus* spp. isolate tested.
