# Supplementary material for: Characterization and Comparison of Enterococcus spp. Isolates from Feces of Healthy Dogs and Urine of Dogs with UTIs
Source: Animals (Basel). 2021 Sep 29;11(10):2845. doi: 10.3390/ani11102845 (PMC8532874; doi:10.3390/ani11102845)
Supplement: Supplementary file 1 [file animals-11-02845-s001.zip › animals-1380815-supplementary.pdf]

Supplementary Material

Table S1. Primers of antibiotic resistance used for PCR in the study.

Determining Resistance to	Target Gene	Sequences (5'- 3')	Annealing Temperature (℃)	Amplicon Size (bp)	References
Glycopeptide	*vanA*	F: GGGAAAACGACAATTGC<br>R: GTACAATGCGGCCGTTA	54	732	Dutka-Malen et al. (1995)
	vanB	F: ATGGGAAGCCGATAGTC<br>R: GATTTCGTTCCTCGACC	54	635	Dutka-Malen et al. (1995)
Penicillin	*pbp4*	F: CAACGAAAGCCTGATGAAATGG<br>R: AATCGCCTTTTTGAGGATCGG	55	1272	Ono et al. (2005)
	pbp5	F: AACAAAATGACAAACGGG<br>R: TATCCTTGGTTATCAGGG	54	779	Obeng et al. (2013)
Aminoglycoside	*aac(6')-Ie-aph(2'')-Ia* (Gentamicin)	F: CAGAGCCTTGGGAAGATGAAG<br>R: CCTCGTGTAATTCATGTTCTGGC	56	348	Vakulenko et al. (2003)
	aph(2'')-Ib (Gentamicin)	F: CTTGGACGCTGAGATATATGAGCAC<br>R: GTTTGTAGCAATTCAGAAACACCCTT	56	867	
	aph(2'')-Ic (Gentamicin)	F: CCACAATGATAATGACTCAGTTCCC<br>R: CCACAGCTTCCGATAGCAAGAG	56	444	
	aph(2'')-Id (Gentamicin)	F: GTGGTTTTTACAGGAATGCCATC<br>R: CCCTCTTCATACCAATCCATATAACC	56	641	
	aph(3')-IIIa (Kanamycin)	F: GGCTAAAATGAGAATATCACCGG<br>R: CTTTAAAAAATCATACAGCTCGCG	56	523	
	ant(4')-Ia (Neomycin, kanamycin, tobramycin and amikacin)	F: CAAACTGCTAAATCGGTAGAAGCC<br>R: GGAAAGTTGACCAGACATTACGAACT	56	294	
	ant(6)-Ia (Streptomycin)	F: CGGGAGAATGGGAGACTTTG<br>R: CTGTGGCTCCACAATCTGAT	55	563	Kobayashi et al. (2001)
	aac(6')-Ii (Gentamicin and other aminoglycosides)	F: TGGCCGGAAGAATATGGAGA<br>R: GCATTTGGTAAGACACCTACG	55	410	
Tetracycline	*tet*(M)	F: GTGGACAAAGGTACAACGAG<br>R CGGTAAAGTTCGTCACACAC	61	406	Malhotra-Kumar et al. (2005)
	tet(L)	F: TGGTGGAATGATAGCCCATT<br>R: CAGGAATGACAGCACGCTAA	61	229	
	tet(O)	F: AACTTAGGCATTCTGGCTCAC<br>R: TCCCACTGTTCCATATCGTCA	61	515	
	tet(K)	F: GATCAATTGTAGCTTTAGGTGAAGG<br>R: TTTTGTTGATTTACCAGGTACCATT	61	155	

Macrolide	*erm*(A)	F: CCCGAAAAATACGCAAAATTTCAT<br>R: CCCTGTTTACCCATTTATAAACG	61	590	Malhotra-Kumar et al. (2005)
	erm(B)	F: TGGTATTCCAAATGCGTAATG<br>R: CTGTGGTATGGCGGGTAAGT	61	745	Malhotra-Kumar et al. (2005)
	mef(A/E)	F: CAATATGGGCAGGGCAAG<br>R: AAGCTGTTCCAATGCTACGC	61	317	Malhotra-Kumar et al. (2005)
	msr(A/B)	F: GCAAATGGTGTAGGTAAGACAACT<br>R: ATCATGTGATGTAAACAAAAT	55	399	Sutcliffe et al. (1996)
	msr(C)	F: TATAACAAACCTGCAAGTTC<br>R: CTTCAATTAGTCGATCCATA	52	410	McDermott et al. (2005)
Lincosamide	*lnuB*	F: CCTACCTATTGTTTGTGGAA<br>R: ATAACGTTACTCTCCTATTC	50	925	Bozdogan et al. (1999)
Streptogramin	*vatE*	F: ACTATACCTGACGCAAATGC<br>R: GGTTCAAATCTTGGTCCG	52	511	Soltani et al. (2000)
	vatD	F: GCTCAATAGGACCAGGTGTA<br>R: TCCAGCTAACATGTATGGCG	55	271	Soltani et al. (2000)
Chloramphenicol	*cat*	F: TAAGGTTATTGGGATAAGTTA<br>R: GCATGRTAACCATCACAWAC	54	340	Hummel et al. (2007)
Int-Tn	(Tn*916*/Tn*1545*)	F: GCGTGATTGTATCTCACT<br>R: GACGCTCCTGTTGCTTCT	50	1028	Doherty et al. (2000)

References

1. Bozdogan B., Berrezouga L., Kuo M.S., Yurek D.A., Farley K.A., Stockman B.J., Leclercq R. A new resistance gene, *linB*, conferring resistance to lincosamides by nucleotidylation in *Enterococcus faecium* HM1025. Antimicrob Agents Chemother. 1999, 43, 925-9.
2. Doherty N.K., Trzcinski P., Pickerill P., Zawadzki C., Dowson G. Genetic diversity of the *tet*(M) gene in tetracycline-resistant clonal lineages of *Streptococcus pneumoniae.* Antimicrob. Agents Chemother. 2000, 44, 2979–2984.
3. Dutka-Malen S., Evers S., Courvalin P. Detection of glycopeptide resistance genotypes and identification to the species level clinically relevant enterococci by PCR. J Clin Microbiol**.** 1995, 33, 24-27.
4. Hummel A.S., Hertel C., Holzapfel W.H., Franz C.M. Antibiotic resistances of starter and probiotic strains of lactic acid bacteria. Appl Environ Microbiol. 2007, 73, 730-739.
5. Kobayashi N., Alam M., Nishimoto Y., Urasawa S., Uehara N., Watanabe N. Distribution of aminoglycoside resistance genes in recent clinical isolates of *Enterococcus faecalis*, *Enterococcus faecium* and *Enterococcus avium*. Epidemiol Infect. 2001, 126, 197-204.
6. Malhotra-Kumar S., Lammens C., Piessens, J., Goossens H. Multiplex PCR for simultaneous detection of macrolide and tetracycline resistance determinants in streptococci. Antimicrob Agents Chemother. 2005, 49, 4798-4800.
7. McDermott P.F., Cullen P., Hubert S.K., McDermott S.D., Bartholomew M., Simjee S., Wagner D.D. Changes in antimicrobial susceptibility of native *Enterococcus faecium* in chicken fed virginiamycin. Appl Environ Microbiol. 2005, 71, 4986-4991.
8. Obeng A.S., Rickard H., Ndi O., Sexton M., Barton M. Comparison of antimicrobial resistance patterns in enterococci from intensive and free range chickens in Australia. Avian Pathol. 2013, 42, 45-54.
9. Ono S., Muratani T., Matsumoto T. Mechanisms of resistance to imipenem and ampicillin in *Enterococcus faecalis.* Antimicrob Agents Chemother. 2005, 49, 2954–2958.

10. Soltani M., Beighton D., Philpott-Howard J., Woodford N. Mechanisms of resistance to quinupristin-dalfopristin among isolates of *Enterococcus faecium* from animals, raw meat, and hospital patients in Western Europe. Antimicrob Agents Chemother. 2000, 44, 433-436.
11. Sutcliffe J., Grebe T., Tait-Kamradt A., Wondrack L. Detection of erythromycin-resistant determinants by PCR. Antimicrob Agents Chemother. 1996, 40, 2562-2566.
12. Vakulenko S.B., Donabedian S.M., Voskresenskiy A.M., Zervos M.J., Lerner S.A., Chow J.W. Multiplex PCR for detection of aminoglycoside resistance genes in enterococci. Antimicrob Agents Chemother. 2003, 47, 1423-1426.

Table S2. Target genes and primers used in this study.

Genes	Encoded Virulence Factor	Primer Sequence 5′–3′	Annealing Temperature	Amplicon length (bp)	Reference
asa1	aggregation substance	F: GCACGCTATTACGAACTATGA<br>R: TAAGAAAGAACATCACCACGA	56	375	Vankerckhoven et al. (2004)
esp	enterococcal surface protein	F: AGATTTCATCTTTGATTCTTGG<br>R: AATTGATTCTTTAGCATCTGG	56	510	Vankerckhoven et al. (2004)
*efaA*fs	*E. faecalis* specific endocarditis antigen	F: GACAGACCCTCACGAATA<br>R: AGTTCATCATGCTGTAGTA	51	705	Eaton & Gasson (2001)
*efaA*fm	*E.faecium* specific surface antigen	F: AACAGATCCGCATGAATA<br>R: CATTTCATCATCTGATAGTA	48	735	Eaton & Gasson (2001)
gelE	gelatinase	F: ACCCCGTATCATTGGTTT<br>R: ACGCATTGCTTTTCCATC	48	419	Lopes et al. (2006)
hyl	hyaluronidase	F: ACAGAAGAGCTGCAGGAAATG<br>R: GACTGACGTCCAAGTTTCCAA	56	276	Vankerckhoven et al. (2004)
cylA	cytolysin activator	F: ACTCGGGGATTGATAGGC<br>R: GCTGCTAAAGCTGCGCTT	56	688	Vankerckhoven et al. (2004)
sgrA	serine–glutamate repeat containing protein A *fms2* or *orf2351*	F: AATGAACGGGCAAATGAG<br>R: CTTTTGTTCCTTAGTTGGTATGA	53	671	Fritas et al. (2018)
pstD	enzyme IID subunit of a putative phos-photransferase system	F: TATCAACGCGATCAAAACGA<br>R: CGTTCGCATACAGCTTTTCA	53	241	Fritas et al. (2018)
orf1481	sugar-binding protein encoded by a ge-nomic island (8.5 kb)	F: GTTTATCAACATGCTAGCCCA<br>R: GCCAATGAGTTAGATGTAGCC	55	437	Fritas et al. (2018)
IS16	*element IS*	F: CATGTTCCACGAACCAGAG<br>R: TCAAAAAGTGGGCTTGGC	55	547	Fritas et al. (2018)

References

1. Eaton T.J., Gasson M.J. Molecular screening of *Enterococcus* virulence determinants and potential for genetic exchange between food and medical isolates. Appl Environ Microbiol. 2001, 67, 1628-1635.
2. Freitas A.R, Tedim A.P, Novais C., Coque T.M., Peixe L. Distribution of putative virulence markers in Enterococcus faecium: towards a safety profile review. J Antimicrob Chemother. 2018, 73, 306-319.
3. Lopes Mde F., Simões A.P., Tenreiro R., Marques J.J., Crespo M.T. Activity and expression of a virulence factor, gelatinase, in dairy enterococci. Int J Food Microbiol. 2006, 112, 208-214.
4. Vankerckhoven V., Van Autgaerden T., Vael C., Lammens C., Chapelle S., Rossi R., Jabes D., Goossens H. Development of a multiplex PCR for the detection of asa1, gelE, cylA, esp, and hyl genes in enterococci and survey for virulence determinants among European hospital isolates of Enterococcus faecium. J Clin Microbiol. 2004, 42, 4473-4479.

Table S3. Origin, species, phenotypic and genotypic profile of each *Enterococcus* spp. isolates tested.

Strain	Source	Species	Resistance pattern	MIC Ampi	HLSR	HLGR	Phenotypic resistance profiles	Genotypic resistance profiles	Biofilm production	Virulence profile
**E244**	Stools	*E. faecalis*	MDR	32	-	+	RD C W N CN S CIP ENR E QD DA OX AMP TE TIG	*pbp4 tet(M) erm(B) aph(3′)-IIIa cat Int-Tn*	MBP	*asa1 efaAfs cylA esp*
**E54**	Stools	*E. faecalis*	MDR	/	+	+	N CN S E QD DA OX TE	*pbp4 tet(M) lnuB erm(B) aph(3′)-IIIa ant(6′)-Ia Int-Tn*	SBP	*gelE asa1 efaAfs cylA esp*
**E53**	Stools	*E. faecalis*	MDR	/	+	+	C F W N CN S E QD DA OX TE	*pbp4 tet(M) lnuB erm(B) aph(3′)-IIIa ant(6′)-Ia cat Int-Tn*	SBP	*asa1 efaAfs cylA esp*
**E220**	Stools	*E. faecalis*	MDR	/	+	+	RD C LZD W N CN S KF CIP ENR E QD DA OX TE TIG	*pbp4 tet(M) lnuB erm(B) aph(3′)-IIIa ant(6′)-Ia Int-Tn*	SBP	*asa1 efaAfs cylA esp*
**E234**	Stools	*E. faecalis*	XDR	/	+	+	RD C LZD W N CN S KF CIP TEC E QD DA OX TE TIG	*tet(M) tet(L) erm(B) aph(3′)-IIIa cat*	WBP	*gelE efaAfs*
**E121**	Stools	*E. faecalis*	MDR	/	+	+	C LZD W N CN S CIP ENR E QD DA OX TE	*pbp4 tet(M) tet(L) lnuB erm(B) aph(3′)-IIIa ant(6′)-Ia cat*	SBP	*gelE efaAfs*
**E165**	Stools	*E. faecalis*	MDR	/	-	+	C LZD F W N CN S CIP ENR QD DA TE TIG	*tet(M) erm(B) Int-Tn*	SBP	*asa1 efaAfs cylA esp*
**E188**	Stools	*E. faecalis*	MDR	/	+	-	RD C LZD W N CN S ENR E QD DA OX TE	*pbp4 tet(M) aph(3′)-IIIa Int-Tn*	MBP	*asa1 efaAfs*
**E232**	Stools	*E. faecalis*	MDR	/	+	+	RD C LZD W N CN S ENR E QD DA OX TE TIG	*pbp4 tet(M) lnuB aph(3′)-IIIa ant(6′)-Ia Int-Tn*	SBP	*gelE asa1 efaAfs cylA esp*
**E270**	Stools	*E. faecalis*	MDR	/	+	-	LZD N CN S QD DA OX TE	*pbp4 tet(M) aph(3′)-IIIa ant(6′)-Ia Int-Tn*	SBP	*gelE asa1 efaAfs cylA esp*
**E218**	Stools	*E. faecalis*	XDR	/	+	-	RD C LZD W N CN S KF CIP ENR E QD DA OX TE TIG	*pbp4 tet(M) lnuB aph(3′)-IIIa ant(6′)-Ia Int-Tn*	SBP	*asa1 efaAfs cylA esp*
**E119**	Stools	*E. faecalis*	MDR	/	+	+	RD C LZD W N CN S CIP ENR E QD DA OX TE TIG	*pbp4 tet(M) tet(L) lnuB erm(B) aph(3′)-IIIa ant(6′)-Ia cat*	MBP	*gelE efaAfs*
**E233**	Stools	*E. faecalis*	XDR	128	+	-	RD LZD W N CN S KF CIP QD DA OX AMP TE TIG	*pbp4 tet(M) aph(3′)-IIIa ant(6′)-Ia*	SBP	*gelE asa1 efaAfs cylA esp*
**E52**	Stools	*E. faecalis*	MDR	/	+	+	N S QD DA OX TE	*pbp4 tet(M) Int-Tn*	SBP	*gelE efaAfs esp*
**1079B**	UTI	*E. faecalis*	MDR	/	-	-	RD N CN S KF CIP ENR QD DA OX	*pbp4*	MBP	*gelE efaAfs*
**654**	UTI	*E. faecalis*	MDR	32	-	-	W S QD DA OX TE	*pbp4 tet(M) Int-Tn*	SBP	*gelE asa1 efaAfs cylA*
**1091**	UTI	*E. faecalis*	MDR	>256	-	-	RD N CN S KF CIP ENR QD DA OX AMP TE	*tet(M)*	MBP	*gelE efaAfs*
**793**	UTI	*E. faecalis*	MDR	64	+	-	RD C N S CIP ENR E QD DA OX TE	*pbp4 tet(M) tet(L) erm(B) aph(3′)-IIIa cat Int-Tn*	WBP	*gelE asa1 efaAfs*
**1079**	UTI	*E. faecalis*	MDR	64	-	-	RD N CN S KF CIP ENR QD DA OX AMP	*pbp4*	SBP	*gelE efaAfs*

**46**	UTI	*E. faecalis*	MDR	8	+	-	C W N CN S KF QD DA OX TE	*pbp4 tet(M) cat Int-Tn*	SBP	*asa1 efaAfs cylA*
**835C**	UTI	*E. faecalis*	MDR	>256	-	-	N CN S KF CIP ENR E QD DA OX TE	*pbp4 tet(M) tet(L) erm(B) Int-Tn*	SBP	*gelE asa1 efaAfs*
**568**	UTI	*E. faecalis*	MDR	16	+	-	C W N CN S KF CIP ENR E QD DA OX TE	*pbp4 tet(M) tet(L) lnuB erm(B) aph(3′)-IIIa ant(6′)-Ia cat Int-Tn*	SBP	*asa1 efaAfs cylA esp*
**260**	UTI	*E. faecalis*	MDR	>256	+	-	N CN S KF CIP ENR QD DA OX	*pbp4*	MBP	*gelE efaAfs*
**323**	UTI	*E. faecalis*	MDR	/	-	-	W N CN S E QD DA OX TE	*pbp4 tet(M) Int-Tn*	SBP	*asa1 efaAfs cylA esp*
**324**	UTI	*E. faecalis*	MDR	/	-	-	W N CN S KF QD DA OX	*pbp4*	WBP	*gelE asa1 efaAfs*
**320**	UTI	*E. faecalis*	MDR	32	-	-	RD N CN S CIP ENR E QD DA OX	*pbp4 erm(B)*	MBP	*gelE asa1 efaAfs*
**359**	UTI	*E. faecalis*	MDR	32	-	-	RD W N CN S KF E QD DA OX TE	*pbp4 tet(M) Int-Tn*	SBP	*asa1 efaAfs cylA esp*
**357B**	UTI	*E. faecalis*	MDR	32	-	-	N CN S KF CIP ENR E QD DA OX AMC AMP TE	*pbp4 erm(B)*	MBP	*gelE asa1 efaAfs*
**415**	UTI	*E. faecalis*	MDR	16	-	-	W N CN S KF E QD OX AMC AMP TE	*pbp4 tet(M) Int-Tn*	SBP	*asa1 efaAfs cylA esp*
**447**	UTI	*E. faecalis*	MDR	>256	-	-	F W S KF CIP ENR E QD DA OX AMC AMP TE	*pbp4 tet(M) Int-Tn*	WBP	*asa1 efaAfs cylA esp*
**E227**	Stools	*E. faecium*	MDR	128	-	-	RD LZD F W N CN S E DA OX AMP	*pbp5 tet(M) lnuB erm(B)) mrs(A/B) mrs(C) aph(3′)-IIIa ant(6′)-Ia aac(6′)-Ii Int-Tn*	WBP	*asa1 efaAfm cylA esp*
**E260**	Stools	*E.faecium*	MDR	>256	-	-	F W N S KF CIP ENR QD OX AMP TE	*tet(M) mrs(A/B) mrs(C) aph(3′)-IIIa ant(6′)-Ia aac(6′)-Ii Int-Tn*	WBP	*gelE asa1 efaAfm cylA esp*
**E169**	Stools	*E. faecium*	MDR	64	-	-	RD C LZD F W N CN S KF CIP ENR QD OX TE TIG	*tet(M) mrs(A/B) mrs(C) aac(6′)-Ii*	WBP	*efaAfm IS16*
**E153**	Stools	*E.faecium*	MDR	64	-	-	RD C LZD F W N S KF CIP ENR E OX AMP TE TIG	*tet(M) tet(L) mrs(A/B) mrs(C) aac(6′)-Ii*	WBP	*efaAfm IS16*
**E251**	Stools	*E.faecium*	MDR	256	-	-	LZD F W N S KF CIP ENR E DA OX AMP	*pbp5 mrs(A/B) mrs(C) aac(6′)-Ii*	WBP	*efaAfm sgrA*
**E236**	Stools	*E.faecium*	MDR	>256	-	-	RD W S KF ENR OX AMP	*mrs(A/B) mrs(C) aac(6′)-Ii*	WBP	*gelE efaAfm*
**E239**	Stools	*E.faecium*	XDR	128	-	-	C LZD F W N CN S KF CIP QD OX AMP TE	*tet(M) mrs(A/B) mrs(C) aac(6′)-Ii Int-Tn*	WBP	*efaAfm IS16*
**E160**	Stools	*E.faecium*	XDR	16	-	-	RD C LZD F W N CN S KF CIP ENR QD OX AMP TE	*tet(M) mrs(A/B) mrs(C) aac(6′)-Ii Int-Tn*	WBP	*efaAfm*
**E257**	Stools	*E.faecium*	MDR	128	-	-	LZD N KF CIP ENR QD DA OX AMP TIG	*mrs(A/B) mrs(C) aac(6′)-Ii*	WBP	*efaAfm IS16*
**E177**	Stools	*E.faecium*	XDR	32	-	-	LZD F W N CN S KF CIP ENR TEC OX AMP TE TIG	*tet(M) mrs(A/B) mrs(C) aac(6′)-Ii Int-Tn*	WBP	*efaAfm IS16*

**E99**	Stools	*E.faecium*	MDR	>256	+	-	F W N CN S KF E DA OX AMP TE TIG	*tet(M) tet(L) lnuB erm(B) mrs(A/B) mrs(C) aac(6′)-Ie-aph(2″)-Ia ant(6′)-Ia aac(6′)-Ii*	WBP	*efaAfm IS16*
**E254**	Stools	*E.faecium*	MDR	>256	+	-	C LZD W N CN S KF CIP ENR E DA OX AMC AMP TE TIG	*tet(L) lnuB erm(B) mrs(A/B) mrs(C) mef(A/E) aph(3′)-IIIa ant(6′)-Ia aac(6′)-Ii*	WBP	*efaAfm sgrA pstD orf1481 IS16*
**E241**	Stools	*E.faecium*	/	256	-	-	RD N S KF DA OX AMP	*pbp5 mrs(A/B) mrs(C) aac(6′)-Ii*	WBP	*efaAfm*
**E238**	Stools	*E.faecium*	MDR	>256	-	-	C LZD F W N S KF OX AMP TE TIG	*mrs(A/B) mrs(C) aac(6′)-Ii*	WBP	*efaAfm*
**E154**	Stools	*E.faecium*	MDR	64	-	-	RD LZD F N S CIP ENR E DA OX AMP TE	*mrs(A/B) mrs(C) aac(6′)-Ii*	WBP	*efaAfm*
**24A**	UTI	*E. faecium*	MDR	>256	-	+	C N CN S KF CIP ENR E QD DA OX AMC AMP TE	*tet(M) tet(L) mrs(A/B) mrs(C) ant(6′)-Ia aac(6′)-Ii cat Int-Tn*	MBP	*asa1 efaAfm cylA sgrA pstD orf1481 IS16*
**1118**	UTI	*E.faecium*	MDR	>256	+	+	W N CN S KF CIP ENR E DA OX AMC AMP TE	*erm(B) mrs(A/B) mrs(C) aac(6′)-Ie-aph(2″)-Ia aph(3′)-IIIa ant(6′)-Ia aac(6′)-Ii*	WBP	*efaAfm sgrA pstD orf1481 IS16*
**399**	UTI	*E.faecium*	MDR	/	-	-	S KF CIP ENR QD DA OX	*mrs(A/B) mrs(C) aac(6′)-Ii*	WBP	*efaAfm orf1481*
**618**	UTI	*E. gallinarum*	MDR	>256	+	+	RD CIP ENR QD DA OX TE	*tet(M) erm(B) aph(3′)-IIIa cat Int-Tn*	MBP	*gelE asa1*
**556B**	UTI	*E. gallinarum*	MDR	<8	-	-	N S CIP ENR E QD DA OX TE	*tet(M) Int-Tn*	MBP	*asa1*
**1034**	UTI	*E. gallinarum*	MDR	64	+	-	N S KF CIP ENR E QD DA OX AMP TE	*tet(M) aph(3′)-IIIa ant(6′)-Ia Int-Tn*	SBP	*esp*

Legend: Stools = isolate from healthy dog stools; UTI = isolate from sick dog urines; AMPI = Minimum Inhibitory Concentration (MIC) for ampicillin expressed in μg/ml; HLSR = High Level Streptomycin Resistance; HLGR = High Level Gentamicin Resistance; RD = rifampicin; C = chloramphenicol; LZD = linezolid; F = nitrofurantoin; W = trimethoprim; N = neomycin; CN = gentamicin; S = streptomycin; KF = cephalothin; CIP = ciprofloxacin; ENR = enrofloxacin; TEC = teicoplanin; E = erythromycin; QD = quinupristin–dalfopristin; DA = clindamycin; OX = oxacillin; AMC = amoxicillin–clavulanic acid; AMP = ampicillin; TE = tetracycline; TIG = tigecycline; WBP = Weak Biofilm Producer; MBP = Moderate Biofilm Producer; SBP = Strong Biofilm Producer.
